# Utilizing the Heterogeneity of Clinical Data for Model Refinement and Rule Discovery Through the Application of Genetic Algorithms to Calibrate a High-Dimensional Agent-Based Model of Systemic Inflammation

**DOI:** 10.3389/fphys.2021.662845

**Published:** 2021-05-19

**Authors:** Chase Cockrell, Gary An

**Affiliations:** Departmen of Surgery, Larner College of Medicine, The University of Vermont, Burlington, VT, United States

**Keywords:** machine learning, agent based modeling, high performance computing, genetic algorithm, biological heterogeneity

## Abstract

**Introduction:** Accounting for biological heterogeneity represents one of the greatest challenges in biomedical research. Dynamic computational and mathematical models can be used to enhance the study and understanding of biological systems, but traditional methods for calibration and validation commonly do not account for the heterogeneity of biological data, which may result in overfitting and brittleness of these models. Herein we propose a machine learning approach that utilizes genetic algorithms (GAs) to calibrate and refine an agent-based model (ABM) of acute systemic inflammation, with a focus on accounting for the heterogeneity seen in a clinical data set, thereby avoiding overfitting and increasing the robustness and potential generalizability of the underlying simulation model.

**Methods:** Agent-based modeling is a frequently used modeling method for multi-scale mechanistic modeling. However, the same properties that make ABMs well suited to representing biological systems also present significant challenges with respect to their construction and calibration, particularly with respect to the selection of potential mechanistic rules and the large number of associated free parameters. We have proposed that machine learning approaches (such as GAs) can be used to more effectively and efficiently deal with rule selection and parameter space characterization; the current work applies GAs to the challenge of calibrating a complex ABM to a specific data set, while preserving biological heterogeneity reflected in the range and variance of the data. This project uses a GA to augment the rule-set for a previously validated ABM of acute systemic inflammation, the Innate Immune Response ABM (IIRABM) to clinical time series data of systemic cytokine levels from a population of burn patients. The genome for the GA is a vector generated from the IIRABM’s Model Rule Matrix (MRM), which is a matrix representation of not only the constants/parameters associated with the IIRABM’s cytokine interaction rules, but also the existence of rules themselves. Capturing heterogeneity is accomplished by a fitness function that incorporates the sample value range (“error bars”) of the clinical data.

**Results:** The GA-enabled parameter space exploration resulted in a set of putative MRM rules and associated parameterizations which closely match the cytokine time course data used to design the fitness function. The number of non-zero elements in the MRM increases significantly as the model parameterizations evolve toward a fitness function minimum, transitioning from a sparse to a dense matrix. This results in a model structure that more closely resembles (at a superficial level) the structure of data generated by a standard differential gene expression experimental study.

**Conclusion:** We present an HPC-enabled machine learning/evolutionary computing approach to calibrate a complex ABM to complex clinical data while preserving biological heterogeneity. The integration of machine learning, HPC, and multi-scale mechanistic modeling provides a pathway forward to more effectively representing the heterogeneity of clinical populations and their data.

## Introduction

Heterogeneity of biological phenotype is an essential characteristic that provides robustness for organisms in variable and ever-changing environments and provides the range of fitness across individuals necessary for natural selection and evolution to function (Csete and Doyle, 2004; Stelling et al., 2006). Accounting for biological heterogeneity, be it in experimental systems or in clinical data, represents one of the most critical challenges to identifying shared and fundamental properties across biological entities (Gough et al., 2017). In addition to the concepts described in Gough et al. (2017), we have previously proposed that multi-scale computational models can serve as focused abstractions of biological systems to enhance the study and understanding of how these systems function; furthermore, enhancing their ability to capture and reflect complex biological heterogeneity can increase their utility as means of generating more robust, generalizable and translatable knowledge (An, 2018). All computational and mathematical models incorporate parameters that help define their behavior; variations of those parameters can be used to represent the heterogeneity seen in the dynamics of the biological systems represented by those models (Cockrell et al., 2020). We have extended this concept to the propose that a “parameter space” that results in recapitulation of bioplausible phenotypes can reflect genetic and epigenetic variation within a population, and assert that the model rule structure, which represents knowledge of the interactions between the components of the biological system, can be optimized to reflect a more accurate interaction network able to capture an increased variation of behavioral phenotypes. Herein we present a method utilizing genetic algorithms (GAs), a machine learning method for complex optimization, to calibrate and refine an agent-based model (ABM) of systemic inflammation to capture the heterogeneity and variability of a clinical data set. This method represents a departure from traditional approaches to calibration and parameterization that generally focus on using “cleaner” data sets with less variation/heterogeneity and/or fitting to a regression that draws a mean through what variation is present in the selected data, a process that can result in over-fit and brittle models. Alternatively, we propose that models (in terms of both parameters and interaction rules) selected for being able to reproduce an entire range of data within a dataset are more robust and generalizable, and therefore able to enhance the translation and applicability of knowledge.

This work focuses on enhancing the utility of ABMs as means of instantiating mechanistic hypotheses (An, 2009). Agent-based modeling is an object-oriented, discrete-event, rule-based, spatially explicit, stochastic modeling method (Bonabeau, 2002). In an ABM, individual agents representing components of the overall system are simulated interacting with each other and with their environment. These interactions are mediated by a pre-defined set of rules, typically derived from the literature and expert knowledge, and often containing stochastic components, to reflect either known probabilistic components in their behavioral rules or epistemic uncertainty regarding how those rules are resolved. As such, ABMs are computational instantiations of mechanistic knowledge regarding the systems being modeled and consequently are often used to simulate complex systems in which the aggregate of individual agent interactions can lead to non-trivial or unintuitive macro-state/system-level behaviors (An et al., 2009). This makes agent-based modeling a powerful technique for representing biological systems; rules are derived from experimentally observed biological behaviors, and the spatially explicit nature of the models give it an inherent ability to capture space/geometry/structure of biological tissue, which facilitates the ability of biomedical researchers to express and represent their hypotheses in an ABM (An, 2009). ABM’s have been used to study and model a wide variety of biological systems (Bonabeau, 2002), from general purpose anatomic/cell-for-cell representations of organ systems capable of reproducing multiple independent phenomena (Cockrell et al., 2014, 2015) to platforms for drug development (An et al., 2011; Cockrell and Axelrod, 2018), and are frequently used to model non-linear dynamical systems such as the human immune system (Baldazzi et al., 2006; Bailey et al., 2007; Cockrell and An, 2017; An, 2018).

In the process of developing an ABM, hypotheses or pieces of existing knowledge are re-framed as *rules* that determine the behavior of the agents when they interact with each and their environment. For example, in the context of a biomedical ABM one of those rules might be the definition of a cytokine signaling pathway, i.e., Tumor Necrosis Factor α (TNFα), a pro-inflammatory cytokine, upregulates Interleukin-10 (IL-10), an anti-inflammatory cytokine. The quantification of the effect that TNFα has on IL-10 in this hypothetical rule is determined by adjusting the parameters associated with that rule during model calibration, a critical step in the development and refinement of an ABM (Bonabeau, 2002; Rogers and Von Tessin, 2004; Bianchi et al., 2007; Windrum et al., 2007; Liu et al., 2017).

### Parameter Space as a Means of Capturing Genetic/Epigenetic/Intrapopulation Variability

**FIGURE 1:**
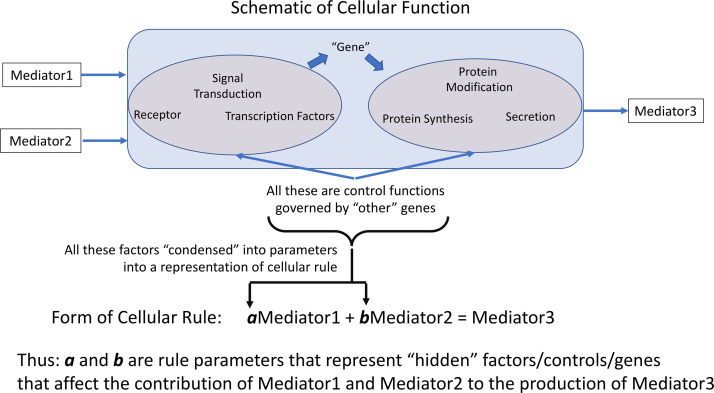
Depiction of how representation of cellular behavioral rules governing the effect and generation of various mediators is accomplished by rule parameters. Cellular rules are presented as input-output relationships for specific cell types; in practice not every mechanistic step is represented in such a rule. The weight of each contributing mediator to the overall function of the cell is represented by the parameters associated within each rule. We pose that these parameters essentially aggregates the influence of non-represented or unknown cellular components on the represented rule. a and b are rule parameters that represent “hidden” factors/controls/genes that affect the contribution of Mediator 1 and Mediator 2 to the production of Mediator 3.

All computational models incorporate parameters within the rules/equations that make up the model. In dynamic mechanistic models, like ABMs, those rules often represent cellular functions and molecular events, such as receptor binding, signaling, gene activation, protein synthesis or secretion (Figure 1). However, the vast majority of mechanism-based computational models do not explicitly represent every component of every step present in the cell; in practice this is nearly functionally impossible at the current time because the sum total of interactions between components, or even the total set of components, is not known. Therefore, essentially all computational models that utilize rules to govern cellular behavior use some degree of abstraction and developer choice in what entities and functions are represented; these choices are often termed the *variables* of the model. These models invariably incorporate sets of parameters/coefficients that reflect the contribution/effect of a particular biological entity/mediator explicitly represented within a model’s rules; these are the *parameters* that modify the variables within a stated rule. We assert that for rules of this type/form the parameters/coefficients represent a concatenation of various mediators, pathways and genes *not explicitly represented* that affect the interaction process represented in the rule (Figure 1), and therefore provide a means of capturing “hidden” control factors (known and unknown) that provide variation across a population of biological entities.

Note that these parameters are an aggregation of a whole series of factors: i.e., the effect of other health factors, such as co-morbidities or age, on the represented rules/functions, unknown mediators or genes, essentially any potential factor than can affect the functional output of the represented rule. Cast in this fashion, the multi-dimensional space of parameters can encompass a range of genetic/epigenetic/functional variability of the type present in a heterogeneous clinical population. We propose that characterizing this parameter space and its associated ensemble of model forms enhances the applicability and generalizability of a model’s rule structure and can avoid “overfitting” and the generation of brittle models. Given the high-dimensional nature of this type of model parameter space we propose to use a machine learning/evolutionary computing optimization method, GAs, in order to generate an *ensemble of parameterizations* able to recapitulate a heterogeneous clinical data set. We would like to emphasize that while GA is an optimization method that will converge to an “optimal” solution, we do not suppose that the optimized solution is necessarily more plausible that the rest of the sufficient parameterizations within the ensemble. Rather, we are utilizing the convergence process of the GA to identify a set of parameterizations sufficient to represent the range of heterogenous clinical data; this ensemble of parameterizations then forms the bioplausible manifestations of simulation model, which can then be used for further studies on disease forecasting (Larie et al., 2020) or therapeutic control discovery (Cockrell and An, 2018; Petersen et al., 2019). Our proposed method is related to how parameter spaces are used to define the behavior of ordinary differential equation (ODE) models, where different fits are used to match different values within a range in a time series of data. However, we believe that the use of ABMs provides an extension of the representational capability of ODE parameter space characterization by the stochastic properties of the ABMs, which reflect intrinsic biological stochasticity, to generate population distributions for individual parameterizations (as opposed to unique deterministic trajectories seen in an ODE).

We also note our attempt to avoid the use of the term “fitting” for this process, a term that brings to mind the way that statistical models are adjusted to match data (though often applied to the calibration of ODE models). Rather than trying to precisely and restrictively identify “fitted” parameterizations, which commonly requires a lossy process by which the heterogeneity of the data is compressed into a mean, we aim to find *sufficient* parameterizations that are able to recapitulate the range of data present. Given how we have defined the role of the parameters in the model (Figure 1) there is no supposition that a “single” parameterization exists within the clinical population, but rather that a population is represented by an ensemble of parameterizations. However, given the epistemic uncertainty associated with all the potential factors that might affect the behavior of the model, it is currently impossible to specify what the distribution across a real population of those parameterizations; the only means we have of determining their plausibility is via the existing data. This strategy is specifically designed to avoid “overfitting,” which we interpret as a failure of generalizability of a particular model when it is exposed to new, additional data; our intent is to preserve and refine the expressiveness of a model’s rule structure with a focus on recapitulating the heterogeneity seen in biological data.

In the sections below we present a method and results that uses the convergence process of GAs to identify an ensemble of parameterizations for an ABM of acute systemic inflammation sufficient to recapitulate the heterogeneity of a clinical data set from burn patients.

## Materials and Methods

### The Model Rule Matrix

In our ABMs the rules and a set of coefficients that quantify the effect of the rules (see Figure 1) are stored in an object which we refer to as the Model Rule Matrix (MRM). In this scheme, specific rules are represented by rows in the matrix; each computationally relevant entity in the model is then represented by the matrix columns. As a simple example, the system of model rule equations for a single cell:

$$M\,1_{t+1}=M\,1_{t}+M\,2_{t}$$

$$M\,2_{t+1}=-M\,1_{t}+M\,3_{t}$$

Would be represented by the matrix:

$$\left[\begin{array}{ccc} 1 & 1 & 0\\ -1 & 0 & 1 \end{array}\right]$$

Where the first column holds the rule coefficients for Mediator 1 (M1), the second column holds the rule coefficients for Mediator 2 (M2), and the third column holds the rule coefficients for Mediator 3 (M3). We note that this is a simplified rule for the purpose of illustration. The matrix is readily decomposable into a one-dimensional vector, upon which we can operate using GAs. The number of rows in the matrix then is equal to the number of rules that it represents, and the number of columns is equal to the number of entities that could potentially contribute to the decision made by their associated rule. Note that if a particular interaction between model components is not represented then the corresponding position within the MRM contains a “0.” Therefore, the MRM presents a compact mathematical representation of the interaction rules present in an ABM.

The resulting product of this work is an ensemble of biologically/clinically plausible model parameterizations, representing a genetically/epigenetically/functionally diverse cohort of *in silico* patients, able to represent a range of heterogeneous experimental or clinical data. In this sense, elements of this work are similar to traditional sensitivity analysis techniques (Cukier et al., 1978; Saltelli et al., 2004, 2008); the primary distinction lies in the fact that these algorithms consider alternate rule configurations (as represented by the conversion of zero to non-zero elements in the MRM), which can change model-parameter sensitivities (Cockrell et al., 2020).

### The Reference Model: IIRABM

In this work, we utilize a previously developed an ABM of systemic inflammation, the Innate Immune Response ABM (IIRABM). Though the IIRABM has been calibrated to simulate blunt trauma and infectious insult, it is an abstract and generalizable (An, 2004; Cockrell and An, 2017) model of human response to injury. Cytokine time series and systemic response varies significantly between both blunt trauma/infectious insult and severe/large surface area burns. In this work, we demonstrate the changes necessary to recalibrate the model from simulating an infectious injury to a caustic and sterile injury. The IIRABM is a two-dimensional abstract representation of the human endothelial-blood interface. This abstraction is designed to model the endothelial-blood interface for a traumatic (in the medical sense) injury and does so by representing this interface as the unwrapped internal vascular surface of a 2D projection of the terminus for a branch of the arterial vascular network. The closed circulatory surface can be represented as a torus, and this two-dimensional surface defines the interaction space simulated by the model. The spatial geometry of the circulatory system and associated organ interfaces are not directly mapped using this scheme. This abstraction reproduces the circulatory topology accessible by the innate immune system and presents a unified means of representing interaction between leukocytes and endothelial surfaces across multiple tissue and organ types. The IIRABM utilizes this abstraction to simulate the human inflammatory signaling network response to injury; the model has been calibrated such that it reproduces the general clinical trajectories of sepsis. The IIRABM operates by simulating multiple cell types and their interactions, including endothelial cells, macrophages, neutrophils, T-lymphocyte subtypes (TH0, TH1, and TH2 cells) as well as their associated precursor cells. Intrinsic biological stochasticity, such as the spatial distribution of cells at initialization or movement direction not governed by chemotaxis and the manifestation of switches governing cellular actions, is represented by the introduction of randomness into the IIRABM; this allows the IIRABM to generate a population distribution of different trajectories from an identical parameterization/initial conditions. The simulated system dies when total damage (defined as aggregate endothelial cell damage) exceeds 80%; this threshold represents the ability of current medical technologies to keep patients alive (i.e., through mechanical organ support) in conditions that previously would have been lethal. The IIRABM is initiated using five parameters representing the size and nature of the injury/infection as well as a metric of the host’s resilience: (1) initial injury size, (2) microbial invasiveness (rate at which infection spreads), (3) microbial toxigenesis (rate at which infection damages tissue), (4) environmental toxicity (amount of spontaneous infectious exposure in the environment, such as an Intensive Care Unit), and (5) host resilience (the rate at which damaged but not dead tissue recovers). These five parameters clearly have correlates in the real world, and yet are nearly inherently un-quantifiable. Therefore, they are treated as dimension-less coordinate axes in which the behavior of the IIRABM exists.

The IIRABM characterizes the human innate immune response through the simulated generation of a suite of biomarkers, including the pro-inflammatory and anti-inflammatory cytokines represented in the IIRABM. At each time step, the IIRABM outputs the total amount of cytokine present for all mediators in the model across the entire simulation. The ordered set of these cytokine values creates a high-dimensional trajectory through cytokine space that lasts throughout the duration of the simulation (until the *in silico* patient heals completely or dies). We note that stochastic effects can play a significant role in simulation dynamics. Model parameterizations used in this work lead to a simulated mortality rate of 50%; in these simulations, identical injuries and initial conditions are given to the model and over time, the trajectories diverge to the point that half of the simulated cohort heals completely and half dies. The fact that the initial conditions are exactly identical means that it is indeed stochasticity, not chaos, that leads to the diverging trajectories. A detailed discussion of this can be found in Cockrell and An (2017).

While the IIRABM successfully simulates the human immune response to injury at a high, overall system level (outcome proportions, time to outcome, etc.), it may not always replicate specific cytokine time series. A cytokine time series is not a single sequence of numerical values; rather, it is a sequence of ranges, indicating significant heterogeneity clinical response to severe burns, within which the cytokine measurements fall for a given patient in the cohort that generated the time series. This heterogeneity is challenging because the magnitude of these ranges is not temporally constant. In order for a computational model to be biologically realistic, it must be able to generate any physiological state which can experienced by the biology that is being simulated and do so with the appropriate frequency. We have previously characterized the shapes of the probabilistic “clouds” of multi-dimensional state space of the IIRABM (Cockrell and An, 2017); these distributions, which are more akin to the range of variable behavior generated by biological systems, are too complex to be represented by a small/simple set of stochastic differential equations with an analytically defined “noise” term. This prompts the need to execute the ABM at large scale in order to more effectively capture the population dynamics structure present in a clinical data set.

### Application of Genetic Algorithms

In this work, we use GA to operate on the IIRABM’s rule set such that it can accurately simulate the cytokine time course and final outcomes for a serious burn injury. As noted in the Introduction, we are employing GA is a non-standard fashion, where rather than seeking a specific optimal parameterization of the MRM we are using the process of convergence of the GA to identify an ensemble of valid parameterizations. Cytokine time series were extracted via inspection from Bergquist et al. (2019). In Bergquist et al. (2019) provide a variety of blood cytokine levels over 15 time points and 22 days for patients which exhibited severe burns over 50% of the surface area of their bodies. The authors observed a mortality rate of 50% for this category of injury.

A GA (Goldberg and Holland, 1988; Fonseca and Fleming, 1993; Haupt and Ellen Haupt, 2004) is a population-based optimization algorithm that is inspired by biological evolution. In a GA, a candidate solution is represented by a synthetic “genome,” which, for an individual, is typically a one-dimensional vector containing numerical values. Each individual in a GA can undergo computational analogs to the biological processes of reproduction, mutation, and natural selection. In order to reproduce, two individual vectors are combined in a crossover operation, which combines the genetic information from two parents into their progeny.

Using this scheme, cytokines produced by a given cell type are held fixed, while the stimuli that lead to the production of that specific cytokine are allowed to vary. This maintains a distinction between the cell and tissue types represented in the model throughout the MRM evolution from the GA.

The candidate genomes which comprise the rule set are then tested against a fitness function which is simply the sum of cytokine range differences between the experimental data and the computational model:

$$F=\sum_{i,t}\left|\max\left(C_{i,t}^{e}\right)-\max\left(C_{i,t}^{m}\right)\right|+k\,\left|R_{e}-R_{m}\right|,$$

where $C_{i,t}^{e}$ represents the normalized blood serum level of cytokine *i* at time point *t* from the experimental data, $C_{i,t}^{m}$ represents the normalized blood serum level of cytokine *i* at time point *t* from the IIRABM, *R*_*e*_ represents the experimentally observed mortality rate, *R*_*m*_ represents the model-generated mortality rate, and *k* is an adjustable parameter to govern the importance of the mortality rate contribution to the fitness function. For the purposes of this work, we consider an optimal solution to be one that minimizes the above fitness function. In order to avoid issues of over-fitting, we held the time points at *t* = 48 h post-burn and *t* = 8 days post-burn back from the evaluation of candidate fitness. Despite this, these time points were well-matched between the *in silico* and *in vivo* experiments.

We note that 50 stochastic replicate simulations of the IIRABM were used to generate simulated ranges, while only 20 patients comprised the clinical data set. The reasoning for this is that the simulated range was not stable using only 20 stochastic replicates; we found that when we ran 50 replicates per parameterization, the simulated cytokine ranges varied only by a few percent. Additionally, we did not have access to individual data points, or distributions at different time points; we only had the maximum and minimum values, and thus were unable to evaluate the effect that additional clinical patients would have had on the observed clinical data range.

Candidate genomes are then selected against each other in a tournament fashion, with a tournament size of 2 [28, 29]. The tournament winners make up the breeding pool, and progenitor genomes are randomely selected and paired. We implement a variant of elitism in that, at the completion of the tournament, the least fit 10% of the candidate progenitors are replaced with the fittest 10% of candidate genomes from the precious generation. Progeny genomes are defined with a uniform crossover operation using a standard continuous formulation (Haupt and Haupt, 2004):

$$C_{1,i}=\beta \,P_{1,i}+\left(1-\beta \right)\,P_{2,i}$$

$$C_{2,i}=\beta \,P_{2,i}+\left(1-\beta \right)\,P_{1,i}$$

Where *C*_*1,i*_ is the value for gene *i* in child 1, *P* is the value for gene *i* in parent 1, and β is a random floating-point number between 0 and 1. After breeding, each child is subject to a random chance of mutation which begins at 1% and increases with each generation.

We employ an elitist strategy by replacing the least fit 10% of the breeding population with the most fit parameterizations. This ensures that our best solutions are not lost due to mutation. Additionally, we utilize two non-standard additions to the GA: the *non-viability criterion* and the *ensemble retainment criterion*. As noted above, the potential parameter space is astronomically large, and the vast majority of those putative parameterizations are in no way biologically viable or plausible; it is therefore desirable to filter these regions of parameter space early in this process. The non-viability criterion immediately rejects any parameterization which leads the model to die before the first clinical time point (3 h post-injury); these are replaced with fitter candidates. In our experience with this model, this non-viability criterion is only activated in the first few generations, as the algorithm quickly finds a focus on viable regions of parameter space. Further, we recognize that any putative parameterization which generates cytokine trajectories that always lie within the cilnically observad range cannot be invalidated by the data, and are therefore biologically plausible; thus, these parameterizations should be retained for inclusion into the final ensemble. As the goal of the fitness function is to obtain maximum coverage over the clinical data range, some of these viable parameterizations may be lost as the population evolves.

The IIRABM was optimized for 250 generations with a starting population size of 1024 candidate parameterizations. The IIRABM was implemented in C++ and the GA was implemented in Python 3; and simulations were performed on the Cori Cray XC40 Supercomputer at the National Energy Research Scientific Computing Center and at the Vermont Advanced Computing Center. Codes can be found at https://github.com/An-Cockrell/IIRABM_MRM_GA. Pseudocode for this procedure is given below:

(1) Initialize starting population, *P*, where each *P*_*i*_ ∈ *P*, is represented by a matrix with elements randomly assigned in the range [−2,2]
(2) REPEAT-UNTIL stopping condition is met (maximum generations or minimum fitness)
(a) BROADCAST candidate parameterizations to available processes
(b) On each process, CALL IIRABM simulation
(c) Determine Fitness, *F*_*i*_
(i) NON-VIABILITY CRITERION: IF *F*_*i*_ > *F*_*c*_ THEN
(1) Discard *P*_*i*_
(2) Replace with *P*_*j≠i*_, where *F*_*j*_ < *F*_*c*_
(d) ENSEMBLE RETAINMENT: Determine Bioplausibility
(i) IF all simulated cytokine values are contained within the range of clinical data, then retain parameterization for inclusion into the ensemble, *E*
(e) GATHER fitnesses to root process
(f) Tournament Selection
(i) Randomly select pairs of parameterizations
(ii) Select fitter parameterization for inclusion into breeding pool *B*
(g) Breeding
(i) Randomly select pairs of parameterizations from *B*
(ii) Generate two progeny parameterizations, where matrix elements are combined using the standard continuous formulation.
(h) Mutation
(i) Set mutation probability, *r*_*m*_ = 0.01 + 0.002*g_*n*_, where *g*_*n*_ is the number of generations completed by the GA
(ii) Generate random number *r*
(iii) IF *r* ≤ *r*_*m*_ THEN randomly select matrix element to mutate, and assign a random value in the range [−2,2]
(i) Check if any fitness has reached the minimum value (0, indicating a single parameterization matches the data perfectly) or the maximum number of generations has been reached.

We note that we ran the algorithm 10 times, all with random seeding parameterizations, and found that, though the initial populations were completely random, the GA converged to the same region of parameter space each time we ran it. This does not preclude the existence of alternate regions, but indicates that, if they exist, their hypervolumes are significantly smaller than the region of parameter space represented by our ensemble population, which is contiguous at the level of resolution that we have used to examine it. Additionally, the simulation never reached a fitness of 0, indicating that a single parameterization of our model cannot explain all the data.

## Results

**FIGURE 2:**
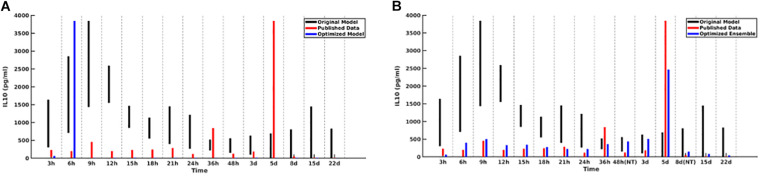
Cytokine ranges are shown for IL-10 for the original model (black), published data (red), and optimized ensemble model (blue). On the left **(A)**, the IL-10 contribution to the fitness function is weighted equally to the other cytokines, with the result that simulated IL-10 levels after 6 h are essentially 0; on the right **(B)**, the IL-10 contribution to the fitness function was doubled.

For the initial attempt with the GA the contributions of each of the five cytokines were weighted equally. This generated an ensemble of sufficient forms of the MRM that produced excellent results for four out of five of the comparison cytokines. However, the GA could not converge well enough to produce MRMs able to generate IL-10 concentrations which matched the literature, with peaking occurring at 6 h post-insult rather than 5 days post-insult, as was seen clinically Figure 2A). As a potential explanation for this inability to replicate IL-10 data we note that in comparison to the other cytokine time series IL-10 showed spikes at *t* = 5 days but is near zero everywhere else, suggesting that a poor fit is more likely when using a fitness function that weights the contributions of each cytokine equally. A candidate MRM parameterization that minimizes IL-10 production over the entire time course would thus contribute less to the overall fitness (in this case, we seek to minimize the fitness function) than a hypothetical parameterization that was 10% off on TNF levels for every time step. In order to address this, we both doubled and tripled the weight of the coefficient to the portion of the fitness function that incorporated IL-10 contribution. Both of these modifications showed similar improvements over the initial fitness function, but neither was significantly better than the other. This leads us to expect that a doubling of the IL-10 contribution to the fitness is sufficient. We display this difference in Figure 2.

**FIGURE 3:**
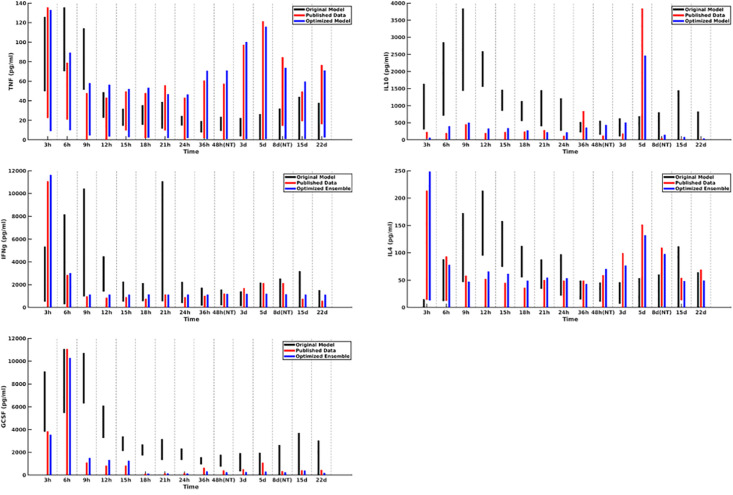
Cytokine ranges are shown for the original model (black), published data (red), and optimized ensemble model (blue) for TNFα (top-left), IL-10 (top-right), IFNγ (center-left), IL-4 (center-right) and GCSF (bottom-left). Ranges for the computational models were generated using 50 stochastic replicates.

A plot of cytokine ranges for 5 cytokines which existed in the clinical data set and were already present in the model at the start of this work (GCSF, TNF-α, IL-4, IL-10, and IFN-γ) is shown in Figure 3. Ranges for the original model, described in Cockrell and An (2017); An (2018), are shown in black; ranges for the published data (Bergquist et al., 2019) are shown in red; and results from the optimized ensemble model are shown in green.

The temporal cytokine dynamics expressed by the optimized IIRABM are significantly modified from its original incarnation. We note that the ensemble models are optimized to match four out of five of the cytokines used in the fitness function to be nearly indistinguishable from the clinical data. We note a slight under-expression of IL-10 at *t* = 5 days post-injury. This discrepancy identifies a weakness in our model when it is being used to simulate burns, namely, that the cellular production of IL-10 is not well enough defined, in that its production is limited to activated macrophages and TH2 helper cells. Given that the IIRABM was developed to represent the innate immune response to traumatic injury, we consider this recalibration to burn injuries to be a success.

**FIGURE 4:**
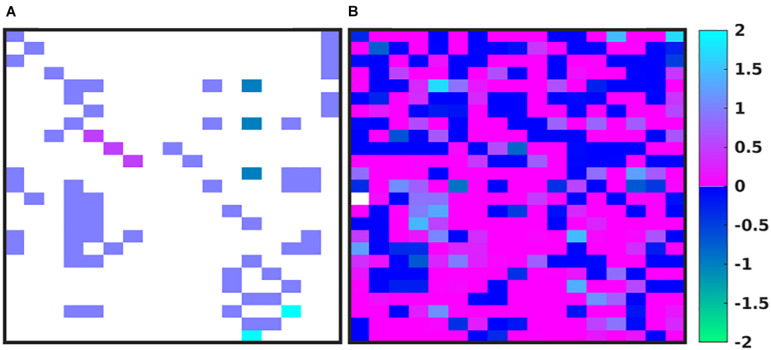
Depictions of the MRM A heatmap of the original rule matrix is shown in panel **(A)**, the optimized matrix representative of the valid ensemble is shown in panel **(B)**. In panels **(A,B)**, the white blocks represent a matrix element with a value of 0 (e.g., no connection); the dark blue to green represents a negative matrix element; the pink to light blue represents a positive matrix element. The optimization process vastly increases the connectivity of the ABM elements (as would be expected in the true biological system).

In Figure 4, we depict the MRM as a heat map of the values (Figures 4A,B) before and at the end of the GA runs. Numerical values for these matrices can be found in the Supplementary Material. Figure 4A shows the MRM values of the original implementation of the IIRABM prior to training; the sparseness of the matrix reflects the necessary abstracting modeling choices made in terms of which rules to represent. Figure 4B shows the “optimized” MRM at the end of the GA runs, noting that while this MRM is the one that most closely matches the *range* of data seen clinically it is representative of the ensemble of MRM able to generate data matching the ranges seen in the clinical data. The optimized matrix has a much more connected structure, and is a dense matrix, as opposed to the sparse original rule matrix. There are not any matrix elements with a value of 0 in the optimized matrix, though there are many elements with comparatively small values. This is an intuitive result and is the intended output based on how the MRM is defined in terms of Figure 1; as all mechanism-based computational models represent a limited and reduced representation of biological reality it is not surprising that there are additional connections needed in order for the model to recapitulate real-world data. As such, this structure of the optimized MRM is similar to what is seen in experimental bioinformatic studies; all of the cytokines in this network appear to be connected to each other, at least to a small degree, while a smaller number of strong connections (which could also be considered correlations) provide the majority of the influence on the system dynamics. The original rule matrix, formatted and with complete labeling, can be found in the Supplementary Material.

**FIGURE 5:**
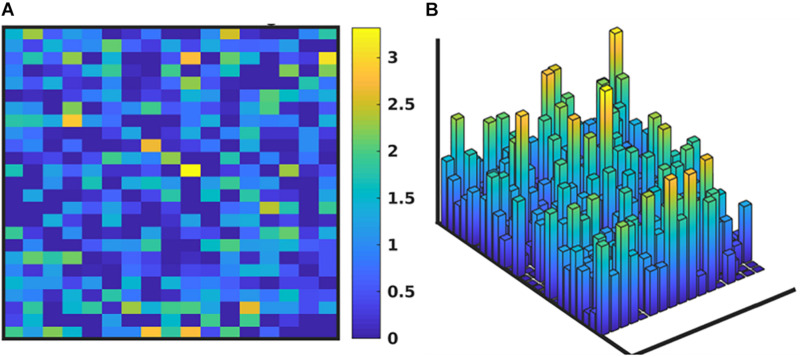
Depiction of the range of values of the MRM for the valid ensemble able to produce data consistent with the clinical data. Panel **(A)** shows the ranges of the MRM values as a heatmap, where dark blue is a range of 0 and yellow indicates a range of 3.42, with a maximal range of 4.0. Panel **(B)** shows this same data as a 3-dimensional bar graph, where the height of each cell reflects the range of the values for each matrix element.

We note that while the process of the GA will lead to convergence to an “optimal” MRM that most closely matches the *range* of data observed clinically, any parameterization which generates a range of data that is encompassed by the clinical data is retained in the ensemble of valid parameterizations. It is this ensemble that is the intended output of the GA process. In Figure 5 we depict the ranges of values of the MRM in the valid ensemble, both as a 2-dimensional heatmap and the same data shown as a 3-dimensional bar graph to aid in visualization of the range of MRM values within the ensemble.

**FIGURE 6:**
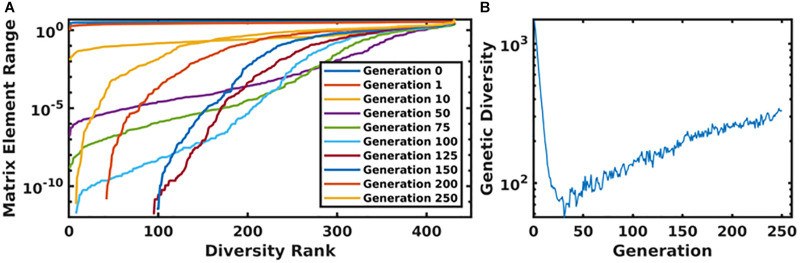
Panel **(A)** displays the ordered matrix element ranges for a variety of time points throughout the genetic algorithm. In this plot, the most diverse generations are represented by a nearly horizontal line at the top of the plot. As the system evolved, this diversity begins to collapse until the increasing mutation rate compensates for the algorithm’s convergence. This is displayed in panel **(B)**, which shows the total diversity of the population as a function of generation number.

In Figure 6, we present the time evolution of the diversity of the simulated population. We define the total diversity of a population to be the sum of the ranges of each matrix element. In Figure 6A, matrix element ranges are ordered from low to high. In the first several generations, diversity is maximized over the entire matrix. As the system evolves toward an optimum parameterization, diversity decreases, and the matrix begins to converge to a single value. In order to combat this, we use a mutation rate that increases as a function of the generation number, which begins to reintroduce diversity into the population. This is seen in Figure 6A, as the matrix element ranges begin to return to a diverse configuration, and more globally in Figure 6B, which plots the total diversity metric as a function of generation number.

## Discussion

The IIRABM rule set utilized in this work contained 432 free and continuous parameters, many of which had highly non-linear or conditional effects on the model-generated cytokine trajectories and outcomes. This high-dimensional parameter space provides an astronomically large set of possible behaviors, of which only a subset are bioplausible. Concurrently, biological objects manifest population-level individual heterogeneity, which means that “bioplausibility” is not a particular trajectory (or mean of trajectories) but rather a set of behaviors and outputs producible by the biological system. Our only guide to this set of behaviors is the range of outputs captured within a data set. The task, then, is to establish a concordance between the range of behaviors represented by a subset of the parameter space of the computational model and the range of outputs seen in the data set and to bound the putative bioplausible parameter space using the data available. The subject of this paper is to present an alternative means of calibrating a computational model to a data set with an emphasis on maintaining the capability to represent the heterogeneity of the data, thereby potentially reflecting critical biological processes that account for the ubiquitous inter-individual variability seen in biological systems.

There are the critical and intertwined issues regarding definition of the fitness function, overfitting, and choice of algorithm. Our utilization of GA was non-standard: while the algorithm sought to optimize the results of the simulation to minimize a fitness function, the discovery of the optimum parameterization was not the actual goal of the work. As our GA traversed the parameter space toward its optimum destination, it gathered all model parameterizations that *were not invalidated* by the available data into the final ensemble. The fitness function was designed such that an optimal solution would minimize the difference between the range of data generated by the model and the range of data observed clinically, but with the explicit aim of defining this bioplausible set rather than finding “a” particular optimal solution.

The design of the fitness function is intimately connected to the concept of overfitting, and some might interpret transition from a sparse rule matrix to a dense rule matrix as the parameter set is optimized as an indication of potential overfitting. This concern stems from the concept of overfitting of statistical models, where the addition of new terms can lead to spurious relationships that may not be present in new data and therefore lead to decreased performance (e.g., failure of generalizability). To some degree this is not the case for mechanism-based dynamic models, where the putative additions to the model represent additional knowledge that (1) has a scientific justification for its addition, (2) theoretically increases the expressiveness (e.g., increased generalizability) of the data and (3) are actually present in the real-world biological object. In addition, from a methodological standpoint, we contend that the traditional concern of overfitting (e.g., failure to generalize) should not be an issue for this approach, according to the following logic:

(1) The primary danger of overfitting is the introduction of spurious elements to the model which would lead to the model’s failure to generalize to new data outside of the data used to train it, ultimately resulting in an invalidation of the model. The primary goal of this work is to generate a diverse population of model parameterizations which are encompassed by the clinical data; when taken in aggregate, and due to the fact that each parameterization generates a range of behavior, this population of parameterizations fills out the range of data observed clinically. While one could claim that a particular added component may not be necessary in order to replicate the data (violation of the concept of parsimony), the addition of such a term cannot be invalidated in comparison to the data.

(2) The introduction of new data *cannot invalidate* individual parameterizations in our ensemble because the introduction of new data can take only two forms: (1) it is either encompassed within the range of the existing data, in which case the previously valid parameterizations are still valid, or (2) new data can be outside the existing range, which does not invalidate any of the previously validated parameterization, but rather suggests an insufficiency in the expressiveness of the previously defined parameter space. In this case an additional search of the parameter space is needed because the current ensemble is insufficiently expressive to explain the heterogeneity of the clinical data and therefore parameterizations that were formerly considered invalid would now be seen to be biologically plausible.

We note that by setting the fitness function to match the published data as exactly as possible we are limiting the targeted degree of heterogeneity to that presented by the relatively small cohort of clinical patients.; the true range of biologically plausible blood cytokine concentrations in undoubtedly larger than what is seen in a small cohort of 20 individuals. In order to obtain a more generalizable model, we propose two alternative approaches to the above presented work: (1) that the fitness function should be configured to over-encompass the available data, with cytokine range boundaries determined by the probability density function (pdf) which governs the experimental data; or (2) synthesize multiple datasets in order to design a fitness with maximum cytokine rage coverage that is still supported by experimental data. Incorporating the shape of the probability density function into the fitness function can be difficult purely as a matter of practicality–often the raw data for human cytokine levels isn’t available, and only the absolute range can be extracted from published manuscripts, and it is also common to see a cohort size that is too small to definitively propose a single pdf which adequately describes the data.

Our approach also involves addressing the limited representation inherent in all computational models. As essentially all mathematical/computational models of biological processes represent some degree of abstraction and are therefore necessarily incomplete, we recognize that the task of model “validation” is more often one of determining the conditions in which a model is “valid” and at what point the model is insufficient. While the employment of the MRM refinement is a means of “encompassing” the uncertainties and “missing” components of the ABM rules, there are still cases where the constraints placed by the choice of rules in the model preclude fitting to particular data points; it is at this point that the model is recognized to be falsified (in the Popperian sense). However, being able to specify where the model fails is extremely useful. In this case, the difficulties in being able to reproduce the trajectories of IL-10 help point to where the IIRABM is insufficient as a representation of the systemic response to burn injury, specifically with respect to the level of representation of anti-inflammatory components. This insight points to the need to incorporate other known anti-inflammatory components into future iterations of the IIRABM.

In future work, we will utilize this method to generate diverse *in silico* cohorts as part of our machine-learning therapeutic discovery workflow (Cockrell and An, 2018; Petersen et al., 2019). We note the importance of *in silico* genetic diversity for therapeutic discovery in Cockrell and An (2018); in this work, we developed a multi-cytokine/multi-time-point therapeutic regimen which decreased the mortality rate from ∼80 to ∼20% for a severe simulated injury. The therapy was discovered using GAs on a single model internal parameterization. When we examined the non-responders, we noted that hyperactivity in specific pathways could manifest negatively, specifically, excess Granulocyte Colony Stimulating Factor activity lead to excess neutrophil recruitment, which instigated a state of perpetual inflammation. Brittle policies/solutions (i.e., those that are not applicable outside of the very specific circumstances used to train them) have long been recognized as a weakness of machine learning research (Holland J.H.(ed.), 1983). In order to overcome this obstacle, data used to train machine-learning algorithms should be sourced as broadly as possible. A useful analogy would be to compare the machine learning experiment to an *in vivo* biological experiment: performing a biological experiment on a set of genetically identical animals will yield less generalizable information than an experiment performed on a set of genetically heterogenous animals.

**FIGURE 7:**
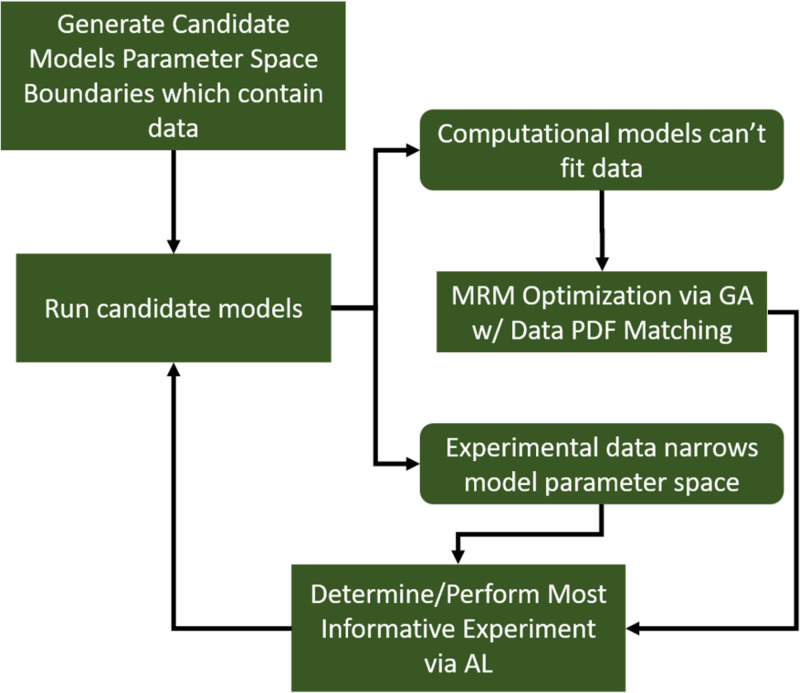
A diagram indicating a hybrid experimental/computational workflow for the automated calibration and validation of ABMs using the MRM scheme. In this workflow, a computational model containing all mechanistic knowledge hypothesized to be relevant to the biological system in question is developed. The range of output for a comprehensive set of viable model parameterizations is determined and compared to biological data. At this point, experimental data can be used to eliminate some of the formerly viable model parameterizations or invalidate the model. In the event the model is invalidated, it can be redesigned/reconfigured to address its shortcomings. After that, the remaining set of putative model parameterizations is investigated to determine which specific parameters contribute the most variability to the model output. These are then the parameters that are selected for further characterization in subsequent biological experiments.

Further, we note that, while we generated a diverse *in silico* patient cohort which generates cytokine trajectories that match clinical data, the diversity is limited by the algorithm. We recognize that by using GA to find a path through parameter space toward some optimum of the fitness function, even though we collect viable parameterizations as the algorithm progresses, they are sampled from a limited region of parameter space. Many of the genes in each individual parameterization end up tightly constrained by the algorithm, while others have a larger range. These latter parameters are those about which the model is most uncertain. Future work will seek to more comprehensively explore the entire parameter space using active learning, similar to Cockrell et al. (2020). Active Learning is a sampling technique used in machine learning in which sampled data is chosen based on how much information it can apply to the machine learning model. A similar approach can be taken in this case. In order to most efficiently update and refine the computational model, experiments should be designed to query the model features that are most uncertain. This approach is illustrated in Figure 7. In this way, GA can play an integral role in the iterative cycle of model refinement and experimentation necessary to construct a high-fidelity generalizable computational model.

## Data Availability Statement

The original contributions presented in the study are included in the article/Supplementary Material, further inquiries can be directed to the corresponding author/s.

## Author Contributions

CC designed the machine-learning workflow, ran simulations, performed data analysis, and contributed to the manuscript. GA designed the initial IIRABM simulation and assisted in the design of the machine-learning workflow and contributed to the manuscript. Both authors contributed to the article and approved the submitted version.

## Conflict of Interest

The authors declare that the research was conducted in the absence of any commercial or financial relationships that could be construed as a potential conflict of interest.
